# Phenotype and Clinical Outcomes of Titin Cardiomyopathy

**DOI:** 10.1016/j.jacc.2017.08.063

**Published:** 2017-10-31

**Authors:** Upasana Tayal, Simon Newsome, Rachel Buchan, Nicola Whiffin, Brian Halliday, Amrit Lota, Angharad Roberts, A. John Baksi, Inga Voges, Will Midwinter, Alijca Wilk, Risha Govind, Roddy Walsh, Piers Daubeney, Julian W.E. Jarman, Resham Baruah, Michael Frenneaux, Paul J. Barton, Dudley Pennell, James S. Ware, Sanjay K. Prasad, Stuart A. Cook

**Affiliations:** a National Heart Lung Institute, Imperial College London, London, United Kingdom; b Cardiovascular Research Centre, Royal Brompton Hospital, London, United Kingdom; c Department of Medical Statistics, London School of Hygiene and Tropical Medicine, London, United Kingdom; d Medical Research Council London Institute of Medical Sciences, Imperial College London, London, United Kingdom; e National Heart Centre, Singapore; f Duke-NUS Medical School, Singapore

**Keywords:** DCM, genetics, CMR, titin, CMR, cardiovascular magnetic resonance, DCM, dilated cardiomyopathy, LGE, late gadolinium enhancement, LMNA, lamin A/C gene, LVMi, indexed left ventricular mass, TTNtv, truncating variant in titin gene

## Abstract

**Background:**

Improved understanding of dilated cardiomyopathy (DCM) due to titin truncation (TTNtv) may help guide patient stratification.

**Objectives:**

The purpose of this study was to establish relationships among TTNtv genotype, cardiac phenotype, and outcomes in DCM.

**Methods:**

In this prospective, observational cohort study, DCM patients underwent clinical evaluation, late gadolinium enhancement cardiovascular magnetic resonance, TTN sequencing, and adjudicated follow-up blinded to genotype for the primary composite endpoint of cardiovascular death, and major arrhythmic and major heart failure events.

**Results:**

Of 716 subjects recruited (mean age 53.5 ± 14.3 years; 469 men [65.5%]; 577 [80.6%] New York Heart Association function class I/II), 83 (11.6%) had TTNtv. Patients with TTNtv were younger at enrollment (49.0 years vs. 54.1 years; p = 0.002) and had lower indexed left ventricular mass (5.1 g/m^2^ reduction; p_adjusted_ = 0.03) compared with patients without TTNtv. There was no difference in biventricular ejection fraction between TTNtv^+/−^ groups. Overall, 78 of 604 patients (12.9%) met the primary endpoint (median follow-up 3.9 years; interquartile range: 2.0 to 5.8 years), including 9 of 71 patients with TTNtv (12.7%) and 69 of 533 (12.9%) without. There was no difference in the composite primary outcome of cardiovascular death, heart failure, or arrhythmic events, for patients with or without TTNtv (hazard ratio adjusted for primary endpoint: 0.92 [95% confidence interval: 0.45 to 1.87]; p = 0.82).

**Conclusions:**

In this large, prospective, genotype-phenotype study of ambulatory DCM patients, we show that prognostic factors for all-cause DCM also predict outcome in TTNtv DCM, and that TTNtv DCM does not appear to be associated with worse medium-term prognosis.

Dilated cardiomyopathy (DCM) affects up to ∼1 in 250 individuals (1) and is the leading global cause of heart transplantation 2, 3. Despite improvements in pharmacological and device-based therapy, clinical outcomes in DCM remain poor, with a 20% 5-year mortality rate 4, 5. As such, there is a pressing need for better mechanistic understanding, improved risk stratification, and identification of novel therapeutic targets for DCM.

Recently, truncating variants in the gene for the sarcomeric protein titin (TTNtv) have been identified as the largest single genetic cause of DCM, found in 10% to 20% of cases 6, 7. However, their phenotypic and clinical significance remains unclear, as the presence and extent of phenotypic and outcome differences in DCM patients with and without TTNtv varies between studies 6, 7, 8, 9.

Our aim in this study was to define the genotype-phenotype correlation of TTNtv and how this was associated with clinical outcome compared with DCM patients without TTNtv.

## Methods

### Study population

The study population comprised 716 patients with DCM confirmed by late gadolinium enhancement (LGE) cardiovascular magnetic resonance (CMR) who were prospectively enrolled in the National Institute for Health Research (NIHR) Royal Brompton Hospital Cardiovascular Biobank project between 2009 and 2015.

This cohort consisted of consecutive referrals to the CMR unit from the dedicated cardiomyopathy service at Royal Brompton Hospital, London, and a network of 30 regional hospitals. Patients were referred for diagnostic evaluation, family screening, or assessment of DCM severity. Within the cardiomyopathy service, all patients without contraindication undergo CMR at presentation. The majority of patients underwent CMR (and were enrolled) at initial diagnosis (n = 381; 53.2%). Of the remainder, 81% (270 of 335) had been diagnosed <2 years before enrollment and CMR confirmation of DCM. All patients provided written informed consent. The study was approved by the regional ethics committee.

### Diagnosis of DCM

DCM was diagnosed based on established CMR criteria of left ventricular dilation and reduced ejection fraction with reference to age- and sex-adjusted nomograms (10) in the absence of known coronary artery disease (presence of subendocardial LGE suggestive of previous myocardial infarction, >50% stenosis in 1 or more major epicardial coronary arteries, or need for previous percutaneous coronary intervention or coronary artery bypass grafting), abnormal loading conditions (uncontrolled hypertension or significant primary valvular disease), or toxin exposure (alcohol consumption in excess of 80 g/day for 5 years meeting criteria for alcoholic cardiomyopathy). A history of well-controlled hypertension or diabetes was documented as comorbidities. A history of any previous chemotherapy was documented. A history of alcohol excess above U.K. government guidelines was documented (21 units/week for men; 14 units/week for women). Although patients with coronary artery disease were excluded, a small proportion of patients had evidence of concomitant myocardial infarction (n = 17; 2.4%).

### Clinical data

Baseline demographic and clinical information was collected for all patients using patient interview, clinical records, electrocardiogram, and family pedigree data. A family history of DCM (DCM in the proband plus at least 1 other family member) (11) or sudden cardiac death (in at least 1 family member up to third-degree relative) (12) was recorded. A history of atrial or ventricular arrhythmia prior to enrollment was documented as previously described (13). Conduction disease was recorded in the presence of any degree of heart block.

### Cardiac magnetic resonance

All patients underwent CMR at 1.5-T (Siemens Sonata or Avanto scanners, Siemens Medical Systems, Erlangen, Germany). Breath-hold steady-state free precession cine images were acquired in 3 long-axis planes and short-axis slices as previously described (5). LGE images were acquired using a breath-hold inversion recovery sequence following administration of 0.1 mmol/kg of gadolinium contrast agent (Magnevist or Gadovist, Bayer, Leverkusen, Germany) as previously described (5). Left ventricular (LV) volumes, function, and mass were measured using a semiautomated threshold-based technique (CMRtools, Cardiovascular Imaging Solutions, London, United Kingdom). Maximum left atrial (LA) volumes were assessed from the 2- and 4-chamber views at ventricular end-systole (14). All volume and mass measurements were indexed to body surface area and referenced to age- and sex-based tables (10). All CMR measurements were performed by operators blinded to genetic and outcome data.

### Genetic sequencing, variant analysis, and confirmation

All patients underwent targeted next-generation sequencing on Illumina or Life Technologies 5500XL platforms using the TruSight Cardio Sequencing kit (Illumina, San Diego, California) (15) or a custom SureSelect^XT^ (Agilent, Santa Clara, California) target capture with equivalent content. Targeted deoxyribonucleic acid libraries were prepared according to the manufacturers’ protocols. Variants were annotated and filtered as previously described (Online Appendix) (6). Further sequencing metric filters were applied including a minimum coverage ≥10, QualbyDepth score >4, and allelic balance ≥0.20.

Truncating variants were defined as nonsense, frameshift, or essential splice site variants. All TTNtv variants were annotated to the titin meta-transcript (ENST00000589042, LRG_391_t1) (Online Table 1). Only rare TTNtv (minor allele frequency in the ExAC [Exome Aggregation Consortium] dataset <0.0001) in constitutively expressed cardiac exons were analyzed (percentage spliced in: >90%) (6). All variants were confirmed with Sanger sequencing or by review of mapped reads in Integrative Genomics Viewer.

For sensitivity analysis, rare (minor allele frequency <0.0001) protein-altering variants (truncating and nontruncating) in the gene-encoding nuclear envelope protein lamin A/C (LMNA) were identified.

### Clinical endpoints

The primary endpoint was a composite of cardiovascular mortality, major arrhythmic events, and major heart failure events. Major arrhythmic events comprised hemodynamically stable and unstable sustained ventricular tachycardia, ventricular fibrillation, appropriate implantable cardiac-defibrillator shock, and aborted sudden cardiac death. Major heart failure events comprised heart transplantation, LV assist device implantation, and unplanned heart failure hospitalization.

Cardiovascular mortality and each of the arrhythmic and heart failure composites were pre-defined secondary endpoints. Endpoints were defined according to the 2014 American College of Cardiology/American Heart Association definitions for cardiovascular endpoints in clinical trials (16) and the 2006 American College of Cardiology/American Heart Association/European Society of Cardiology guidelines for management of patients with ventricular arrhythmias (17), and are described in detail in Online Table 2.

### Follow-up data collection and adjudication

Follow-up data was collected from primary and hospital care records and patient questionnaires. Survival status was also identified using the U.K. Health and Social Care Information Service to ensure that no deaths were missed. Death certificates and post-mortem reports were obtained where applicable. All primary endpoint events were adjudicated by an independent committee of 3 senior cardiologists (J.J., R.B., M.F.) with expertise in electrophysiology, heart failure management, and clinical trial adjudication, who were blinded to imaging and genetic data. Follow-up time was truncated at 10 years given the reduced number of individuals with follow-up beyond 10 years.

### Statistical analysis

Continuous normally distributed data are expressed as mean ± SD, and non-normally distributed data as median and interquartile range (IQR). Continuous variables were compared using the Mann-Whitney test. Categorical data are expressed as number and percentages, and compared using the Fisher exact test. Multivariable linear regression analysis was used to evaluate predictors of LV mass and LV ejection fraction.

Outcome analysis was conducted on the subset of patients (n = 604) who were recruited to the study prior to December 31, 2014, to allow at least 1 year of follow-up time for all recruited subjects. For the survival analysis, event-free survival was calculated from the date of study entry to the date of the first event in the composite endpoint. Data for all patients who were last known to be alive, or who had died after December 31, 2015, were censored on December 31, 2015.

The Kaplan-Meier method was used to estimate cumulative freedom from each endpoint, and the log-rank statistic was used to test the null hypothesis that there was no difference between groups in the probability of an event at any time point.

An optimized baseline model predicting the primary endpoint was built using Cox proportional hazard modelling evaluating clinical and demographic variables (Online Appendix and Online Table 8). The primary analysis was to evaluate association of TTNtv with the primary endpoint, on univariable and multivariable analysis.

All statistical analyses were conducted in the R environment, version 3.3.1 (R Foundation for Statistical Computing, Vienna, Austria). Anonymized data from participants who consented to data sharing will be made available to researchers through request to the corresponding author.

## Results

### Baseline characteristics

The final study population consisted of 716 patients with a clinical and imaging diagnosis of DCM confirmed by CMR. There were 469 men (65.5%), and the majority of the cohort was Caucasian (n = 608; 84.9%). Mean age at recruitment was 53.5 ± 14.3 years (range 11.5 to 88.4 years). The majority of patients were in New York Heart Association functional class I (n = 300; 41.9%) or II (n = 277; 38.7%) at enrollment, reflecting the ambulatory status of the patients in this cohort. A summary of demographics, clinical factors and baseline medication is shown in Online Table 3.

Imaging characteristics of the DCM cohort are shown in Online Figure 1 and Online Table 4. Overall, mean left ventricular ejection fraction (LVEF) was 39.0 ± 12.5% (median 40%; IQR: 30% to 49%). In line with other DCM cohorts, midwall myocardial fibrosis, detected through LGE-CMR, was found in 250 (34.9%) patients—mainly localized in the septum (n = 188; 75.2%) (Online Table 4).

### Curation of titin truncating variants

**Figure 1:**
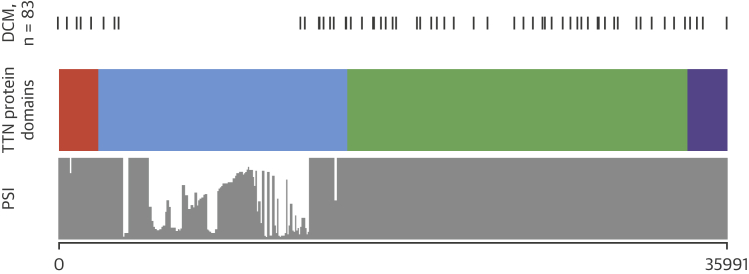
Position of TTNtv Variants According to Protein Domains and PSI **Orange** indicates Z disc; **blue** indicates I band; **green** indicates A band; and **purple** indicates M line. DCM = dilated cardiomyopathy; PSI = percentage spliced in; TTNtv = truncating variants in the titin gene.

TTNtv in constitutively expressed cardiac exons were found in 83 patients (11.6%). The variants are listed in Online Table 5. The position of the TTNtv and protein domains is shown in Figure 1.

### TTNtv cardiomyopathy phenotype

Patients with TTNtv were younger at study recruitment compared with patients without TTNtv (49.0 years vs. 54.1 years; p = 0.002). There was no significant difference in sex (p = 0.27) or ethnicity (p = 0.39) between groups (Table 1). As expected for a genetic disorder, patients with TTNtv were more likely to have a family history of DCM (Table 1). Amongst the TTNtv patients with a family history of DCM (n = 26), 22 in this cohort were probands and 4 were affected family members. There was no difference in the frequency of a family history of sudden cardiac death between groups (Table 1). There was a lower incidence of left bundle branch block (LBBB) at baseline in patients with TTNtv compared with patients without TTNtv (Table 1).

**Table 1:** Baseline Demographics in DCM Patients Stratified by TTNtv Status

	TTNtv Absent (n = 633)	TTNtv Present (n = 83)	p Value
Age at baseline, yrs	54.1 ± 14.3	49.0 ± 13.5	0.002
Male	410 (64.8)	59 (71.1)	0.27
Caucasian	533 (84.2)	75 (90.4)	0.39
Family history of DCM	87 (13.7)	26 (31.3)	<0.001
Family history of sudden cardiac death	95 (15.0)	12 (14.5)	1.0
History of peripartum cardiomyopathy	6 (0.9)	1 (1.2)	0.58
History of chemotherapy	32 (5.1)	2 (2.4)	0.41
History of moderate alcohol excess∗	98 (15.5)	13 (15.7)	1.0
NYHA functional class†			0.17
1	261 (43.1)	39 (49.4)	
2	245 (40.4)	32 (40.5)	
3	94 (15.5)	6 (7.6)	
4	6 (1.0)	2 (2.5)	
Hypertension	203 (32.1)	10 (12.0)	<0.001
Diabetes mellitus	79 (12.5)	8 (9.6)	0.57
Beta-blocker use	444 (70.1)	60 (72.3)	0.80
ACE inhibitor or ARB use	503 (79.5)	64 (77.1)	0.67
Aldosterone antagonist use	226 (35.7)	31 (37.3)	0.81
Diuretic use	287 (45.3)	41 (49.4)	0.49
Conduction disease	217 (34.3)	18 (21.7)	0.03
Left bundle branch block	179 (28.5)	8 (9.8)	<0.001
History of atrial fibrillation	140 (22.1)	23 (27.7)	0.27
History of nonsustained ventricular tachycardia	67 (10.6)	16 (19.3)	0.03
History of sustained ventricular tachycardia	14 (2.2)	1 (1.2)	1.0

Age is summarized as mean ± SD and compared with the Mann-Whitney test. The remaining categorical variables are summarized as n (%) and compared using the Fisher exact test.

ACE = angiotensin-converting enzyme; ARB = angiotensin receptor blocker; DCM = dilated cardiomyopathy; NYHA = New York Heart Association; TTNtv = truncating variants in the titin gene.

∗ Patients with a history of alcohol consumption in excess of the U.K. government weekly “sensible limits.” Patients meeting criteria for alcoholic cardiomyopathy were excluded from the cohort.

† Missing data in 31 patients (4.3%).

There was no significant difference in biventricular ejection fraction in patients with and without TTNtv (median LVEF TTN^+/−^: 39.0% [IQR: 27.0% to 49.0%] vs. 40.0% [IQR: 30.0% to 49.0%]; p = 0.48; median RVEF TTN^+/−^: 53.0% [IQR: 44.0% to 62.0%] vs. 54.0% [IQR: 44.0% to 61.0%]; p = 0.65) (Table 2). There were no significant differences in RV stroke volume or cardiac chamber dimensions (left and right ventricular volumes or left atrial volume) between patients with TTNtv and patients without TTNtv (Table 2). Patients with TTNtv had a minor reduction in indexed LV stroke volume compared with patients without TTNtv (median TTNtv^+/−^ 43.6 ml/m^2^ vs. 47.8 ml/m^2^; p = 0.007).

**Table 2:** Baseline CMR Variables in DCM Cohort Stratified by TTNtv Status

	TTNtv Absent (n = 633)	TTNtv Present (n = 83)	p Value
LV ejection fraction, %	40.0 (30.0–49.0)	39.0 (27.0–49.0)	0.48
Indexed LV end-diastolic volume, ml/m^2^	118.5 (103.5–143.8)	111.1 (100.6–136.4)	0.14
Indexed LV end-systolic volume, ml/m^2^	68.9 (53.8–96.7)	67.4 (51.0–100.1)	0.52
Indexed LV stroke volume, ml/m^2^	47.8 (13.3)	43.6 (12.8)	0.007
Indexed LV mass, g/m^2^	87.4 (73.6–106.5)	81.3 (69.4–96.8)	0.02
RV ejection fraction, %	54.0 (44.0–61.0)	53.0 (44.0–62.0)	0.65
Indexed RV end-diastolic volume, ml/m^2^	84.7 (70.5–101.8)	84.9 (70.1–99.4)	0.61
Indexed RV end-systolic volume, ml/m^2^	40.0 (28.8–53.0)	41.0 (28.0–51.5)	0.96
Indexed RV stroke volume, ml/m^2^	44.2 ± 12.7	41.7 ± 13.1	0.10
Indexed left atrial volume, ml/m^2^	54.1 (43.9–68.4)	57.2 (42.4–70.6)	0.79
Maximum LV wall thickness, mm	10.0 ± 2.2	8.8 ± 1.8	<0.001
Mean septal wall thickness, mm	7.9 ± 1.9	7.2 ± 1.5	0.002
Mean lateral wall thickness, mm	5.6 ± 1.6	5.0 ± 1.2	0.001
Midwall late gadolinium enhancement	224 (35.4)	26 (31.3)	0.54
Bystander myocardial infarction (in patients with normal coronary artery status)	16 (2.5)	1 (1.2)	0.62
Mitral regurgitation			0.48
None	340 (53.7)	49 (59.0)	
Mild	210 (33.2)	23 (27.7)	
Moderate	66 (10.4)	11 (13.3)	
Severe	15 (2.4)	0 (0.0)	

Values are median (interquartile range), n (%), or mean ± SD. Between group comparisons were made using Mann-Whitney or Fisher exact test as appropriate.

CMR = cardiac magnetic resonance; LV = left ventricular; RV = right ventricular; other abbreviations as in Table 1.

**Figure 2:**
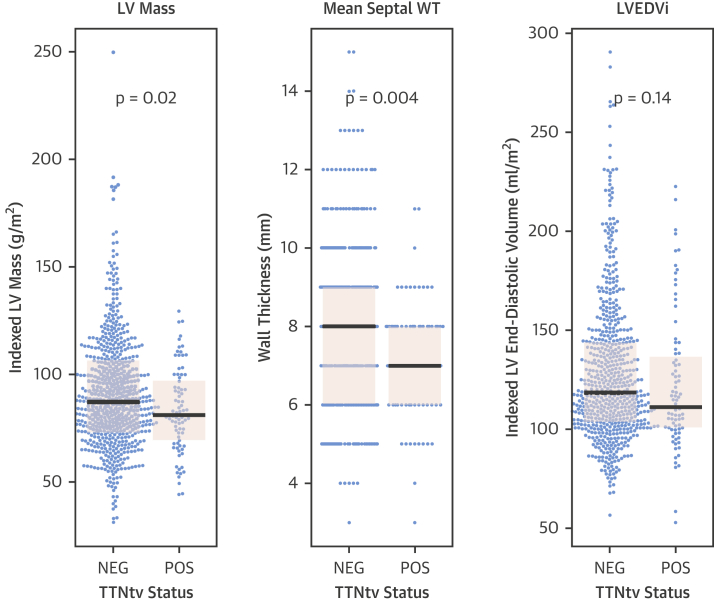
LV Hypertrophy in TTNtv DCM Beeswarm and boxplot (**black bars** indicate median, **pink shaded boxes** indicate interquartile range) of indexed left ventricular (LV) mass, mean septal wall thickness (WT), and indexed LV end-diastolic volume (LVEDVi) stratified by titin status, showing that patients with TTNtv have lower indexed LV mass and thinner LV walls in the absence of evidence of differences in LV dilatation. Between-group comparisons are made using the Mann-Whitney test. NEG = negative; POS = positive; other abbreviations as in Figure 1.

Notably, patients with TTNtv had thinner LV walls (mean maximum LV wall thickness TTNtv^−^ = 10.0 ± 2.2 mm; TTNtv^+^ = 8.8 ± 1.8 mm); p < 0.001) and lower indexed left ventricular mass (LVMi) (mean LVMi TTNtv^−^ = 87.4 ± 26.4 g/m^2^; TTNtv^+^ = 81.3 ± 19.6 g/m^2^; p = 0.02) in the absence of statistically significant differences in indexed LV end-diastolic volume (mean TTNtv^−^ = 127.7 ± 35.8 ml/m^2^, TTNtv^+^ = 122.5 ± 34.5 ml/m^2^; p = 0.14), compared with patients without TTNtv (Figure 2, Table 2).

**Figure 3:**
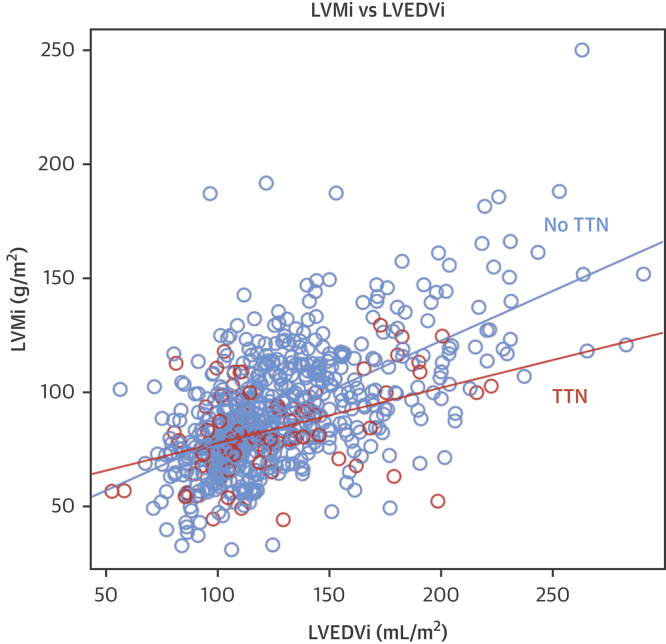
Relationship Between LVMi and LVEDVi in Patients With and Without TTNtv **Orange circles** are patients with TTNtv. **Blue circles** are patients without TTNtv. The regression slope of indexed left ventricular mass (LVMi) ∼ LVEDVi (LVMi increase per 1-ml/m^2^ increase in LVEDVi) is shown for patients with TTNtv (**orange line:** 0.26g/m^2^) and without TTNtv (**blue line:** 0.42g/m^2^). Typically, LVMi increases in line with increasing LVEDVi, but this effect is reduced in TTNtv DCM (p = 0.006). Abbreviations as in Figures 1 and 2.

Typically, LVMi increases in line with increasing indexed LV end-diastolic volume, but this effect is reduced in TTNtv DCM (increase in LVMi per 1-ml/m^2^ increase in indexed LV end-diastolic volume: 0.26 g/m^2^ for patients with TTNtv, 0.42 g/m^2^ for patients without TTNtv; p = 0.006) (Figure 3). However, patients with TTNtv were less likely to have a history of hypertension (12.0% vs. 32.1%; p < 0.001) (Table 1); therefore, a linear regression analysis was performed to evaluate predictors of LV mass.

A baseline regression model predicting LV mass was built as outlined in the Online Appendix and Online Table 9. On univariable linear regression analysis, the presence of TTNtv was associated with a 7.8-g/m^2^ reduction in LVMi (95% confidence interval: −13.7 to −1.8 g/m^2^; p = 0.01). On multivariable analysis, TTNtv was associated with lower LVMi after adjusting for the covariates in the baseline model, including hypertension, with an estimated 5.1-g/m^2^ lower LVMi (adjusted p = 0.03) (Table 3).

**Table 3:** Linear Regression Analysis Evaluating Predictors of Left Ventricular Mass

	Unadjusted Analysis			Adjusted Analysis		
	Estimated Change in LVMi (g/m^2^)	95% Confidence Interval	p Value	Estimated Change in LVMi (g/m^2^)	95% Confidence Interval	p Value
Male	17.7	13.9 to 21.5	<0.0001	12.7	9.5 to 15.8	<0.0001
History of hypertension	6.8	2.7 to 11.0	0.001	7.6	4.3 to 10.8	<0.0001
LVEDVi (per 1-ml/m^2^)	0.42	0.37 to 0.46	<0.0001	0.39	0.34 to 0.43	<0.0001
Presence of TTNtv	−7.8	−13.7 to −1.8		−5.1	−9.7 to −0.4	0.03

LVEDVi = indexed left ventricular end-diastolic volume; LVMi = indexed left ventricular mass; TTNtv = truncating variants in the titin gene.

There was no significant difference in the pattern of late gadolinium enhancement seen in DCM patients with TTNtv compared with patients without TTNtv. Specifically, there was no significant difference in the presence or location of LV midwall fibrosis, an important prognostic marker detected in one-third of cases with DCM (Table 2).

### Positional effects of TTNtv on cardiac endophenotypes

**Figure 4:**
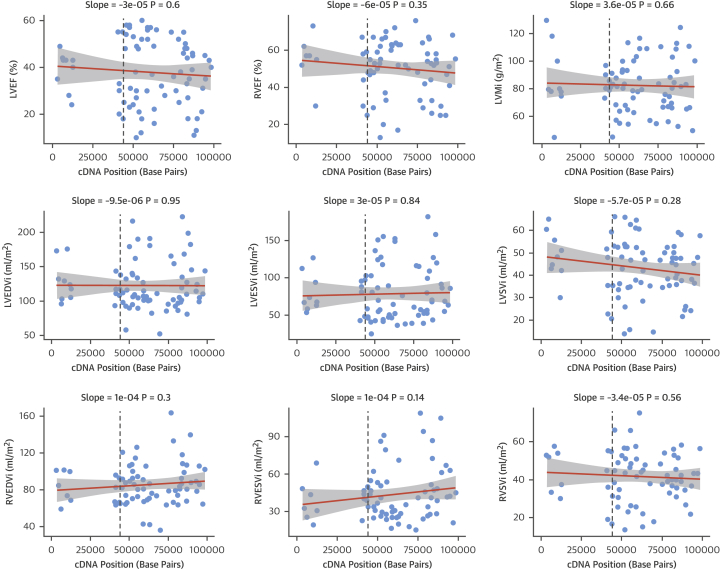
Relationship Between TTNtv Location and Cardiac Endophenotypes Assessed by CMR in the Cohort The TTNtv location, defined by the complementary deoxyribonucleic acid (cDNA) position, is plotted on the x-axis (units = base pairs). A regression line with 95% confidence intervals is shown for each variable, and the slope and p value of the regression is shown **above each plot**. Only variants in constitutive exons are plotted. The **dashed lines** indicate the expected position of the Cronos promoter (upstream of exon 240, numbered according to the LRG391_t1 meta-transcript). CMR = cardiovascular magnetic resonance; LVEF = left ventricular ejection fraction; LVESVi = left ventricular indexed end-systolic volume; LVSVi = left ventricular indexed stroke volume; RVEDVi = right ventricular indexed end-diastolic volume; RVEF = right ventricular ejection fraction; RVESVi = right ventricular indexed end-systolic volume; RVSVi = right ventricular indexed stroke volume; other abbreviations as in Figures 1, 2, and 3.

Given that TTNtv variant location has previously been suggested to influence phenotype in a small cohort (n = 42; of which 36 patients are included in this cohort) (6), we explored this further in this larger cohort. On univariable analysis of TTNtv and LVEF, RVEF, LVMi, and biventricular volumes, there was no significant relationship between TTNtv location and severity of the cardiac phenotype (Figure 4).

### Clinical outcomes in DCM

In total, 604 patients were followed up for a median of 3.9 years (IQR: 2.0 to 5.8 years). A total of 78 patients (12.9%) met the primary composite endpoint, with 24 (4.0%) cardiovascular deaths, 24 (4.0%) patients meeting the arrhythmic secondary endpoint, and 50 (8.3%) patients meeting the heart failure secondary endpoint (Online Tables 6 and 7).

A baseline Cox model predicting the primary outcome was built as outlined in the Online Appendix. The final baseline model predicting the primary endpoint consisted of LVEF, presence of midwall fibrosis LGE, indexed left atrial volume, and sustained ventricular tachycardia (Table 4).

**Table 4:** Baseline Cox Proportional Hazard Model Predicting the Primary Endpoint

	Unadjusted Analysis			Adjusted Analysis		
	HR	95% CI	p Value	HR	95% CI	p Value
Left ventricular ejection fraction, per 10%	0.70	0.57–0.81	<0.0001	0.70	0.58–0.84	0.0002
Indexed left atrial volume, per 10 ml/m^2^	1.11	1.07–1.16	<0.0001	1.11	1.06–1.17	0.00001
Midwall fibrosis late-gadolinium enhancement present	2.39	1.53–3.74	0.0001	2.26	1.41–3.64	0.0008
Sustained ventricular tachycardia present	4.70	2.04–10.85	0.0003	3.24	1.28–8.24	0.01

CI = confidence interval; HR = hazard ratio.

### Titin and clinical outcomes: Primary endpoint

**Figure 5:**
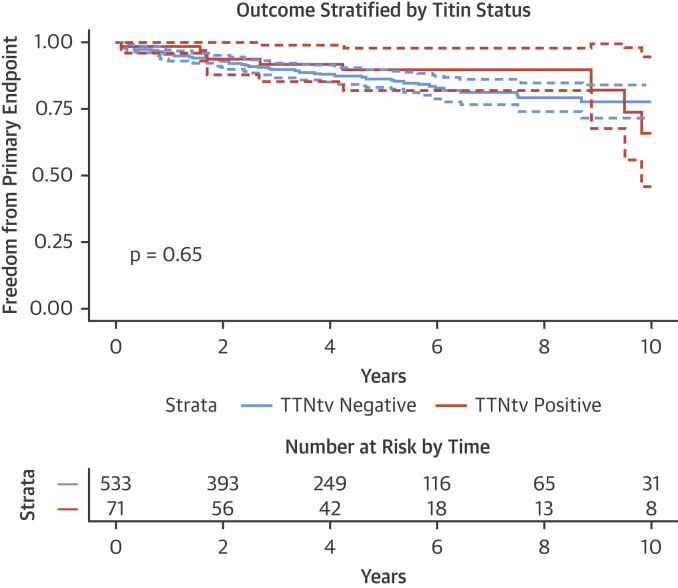
Survival Curves Comparing Freedom From the Primary Endpoint in Patients With and Without TTNtv Curves are compared using the log-rank test. Confidence intervals are shown as **dashed lines**. Follow-up time is shown as years since study enrollment. There is no significant difference between the 2 groups for the primary endpoint on unadjusted analysis. TTNtv = truncating variants in the titin gene.

Of the 604 patients in the outcome cohort, 71 patients had TTNtv (11.8%). Of these, 9 patients (12.7%) with TTNtv met the primary endpoint compared with 69 of 533 patients (12.9%) without TTNtv. Survival curves comparing the freedom from the primary endpoint for patients with and without TTNtv are shown in Figure 5.

On univariable Cox proportional hazard modelling, the presence of a TTNtv has a hazard ratio (HR) of 0.81 (95% confidence interval: 0.41 to 1.63; p = 0.56) for the primary endpoint. On multivariable analysis (adjusting for variables in Table 4), the presence of a TTNtv was not associated with the primary endpoint (HR: 0.92; 95% confidence interval: 0.45 to 1.87; p = 0.82). Therefore, in this cohort, there was no evidence to suggest that TTNtv was a significant prognostic indicator.

### Titin and clinical outcomes: Secondary endpoints

There was no significant difference between TTNtv^+^ and TTNtv^−^ DCM patients for the secondary arrhythmic and heart failure endpoints and cardiovascular mortality (Online Figure 2).

### Sensitivity analysis: LMNA variants

Currently, LMNA variants are the only clinically actionable DCM genetic variants; therefore, sensitivity analysis was performed after exclusion of 8 patients with rare variants in LMNA, of whom 4 met the primary endpoint. The results from the unadjusted and adjusted HR for TTNtv on the primary endpoint were very similar (unadjusted HR: 0.85; p = 0.64; adjusted HR: 0.92; p = 0.83).

## Discussion

In this study of 716 patients with DCM, we combined TTN sequencing with detailed cardiac phenotyping using CMR to define genotype–phenotype correlations and their associations with outcomes in TTNtv cardiomyopathy. As the commonest genetic cause of DCM 1, 6, 7, improved understanding of the clinical manifestations of TTNtv cardiomyopathy offers scope for refined patient stratification by genotype.

We demonstrate that DCM due to TTNtv is associated with a blunted hypertrophic response, highlighting possible disease mechanisms. We show that TTNtv DCM has a similar adverse event profile to non–TTNtv DCM (Central Illustration). This data may help inform clinical management in DCM. Finally, this study highlights an improvement in DCM outcomes overall when compared with previous registry and study data 5, 18, 19.

**Central Illustration:**
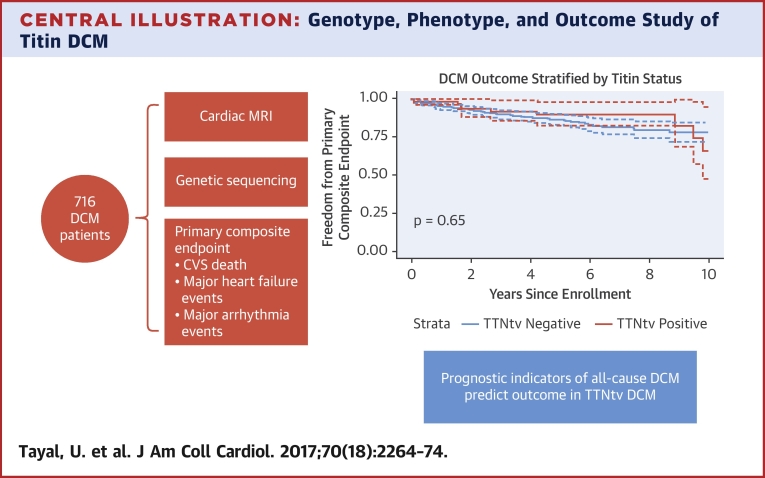
Genotype, Phenotype, and Outcome Study of Titin DCM Truncating variants in the titin gene (TTNtv) are the largest single genetic contributor to dilated cardiomyopathy (DCM). In this large, prospective, genotype-phenotype study of ambulatory DCM patients, we show that prognostic indicators for all-cause DCM also predict outcomes in TTNtv DCM, and that TTNtv DCM does not appear to be associated with a substantially worse medium-term prognosis, despite patients presenting at a younger age. CVS = cardiovascular; MRI = magnetic resonance imaging.

### Novel insights into the TTNtv phenotype

In this study, we find that the functional cardiac phenotype in DCM patients with TTNtv is equivalent to that of patients without TTNtv, albeit that TTNtv DCM is associated with a lower LVMi.

Previous work from our group in a small allelic series of TTNtv DCM cases (n = 42) showed that the TTNtv that affected the protein distally toward the Cʹ terminus were associated with more severe cardiac endophenotypes. A recent zebrafish study identified an internal promoter in the distal I band (just before exon 240) for a short titin isoform, named *Cronos*, that is active in mouse and human hearts and, when disrupted, resulted in a severe phenotype (20). In this study, we demonstrated that the cardiac phenotype of TTNtv is not accounted for by variant position, including *Cronos* disruption.

We did, however, find that TTNtv DCM is associated with thinner LV walls and lower LV mass in the absence of evidence of significant differences in LV dilation. This effect remained after controlling for important clinical covariables. Although the magnitude of difference in wall thickness and LV mass are not clinically informative and cannot be used to predict genotype based on phenotype, they may offer a potential insight into the pathogenesis of TTNtv DCM. At the sarcomeric level, titin plays a key role in the mechanotransductive response of the cardiomyocyte and regulation of cardiac hypertrophy. Pathological hypertrophy and ventricular dilation and dysfunction develop in response to biological stressors, such as neurohormonal activation or myocyte injury. Pathological hypertrophy is thought to develop from an adaptive compensatory response to a maladaptive state (21). The mammalian target of rapamycin (mTOR) is an evolutionary conserved kinase that can modulate cardiac hypertrophy (22), and increased mTORC1 signaling is an adaptive response to cardiac stress in rat models. We have recently shown that rats with TTNtv had elevated mTORC1 signaling at baseline but were not able to increase signaling when stressed (23). This may offer a biological explanation for the blunted hypertrophic response observed in human TTNtv DCM, although the mechanistic link between TTNtv and mTORC activation remains to be established.

### Prevalence of TTNtv in DCM

The prevalence of TTNtv in this cohort (∼12%) is lower than in previous studies, which showed a prevalence of up to 25% in familial DCM cases (7). However, TTNtv frequency ranged from ∼8% to 40% across the 3 clinical cohorts in the study by Herman et al. (7), with the highest frequencies observed in patients evaluated for cardiac transplantation or with severely impaired function. Therefore, it is plausible that TTNtv are enriched in severe/end-stage DCM cohorts, and the prevalence of ∼12% that we observe is a more accurate reflection of the frequency of TTNtv in an unselected and ambulant DCM population. This is supported by the 14% frequency of TTNtv observed in an independent cohort of DCM patients referred for clinical genetic testing (24).

### TTN and sex

Previous studies have reported conflicting outcomes for TTNtv DCM stratified by sex 6, 7, 8, 9. In our cohort of TTNtv DCM patients, 73% were male. The sex distribution is interesting because DCM is known to have a 3:1 male to female ratio; yet, one might expect an autosomal dominantly inherited genetic form of DCM to have a 1:1 male to female ratio. This suggests that additional factors (modifiers) may be important for the development of phenotype (penetrance) in individuals with TTNtv and that the presence of TTNtv alone may not be sufficient.

### Clinical outcomes in TTNtv cardiomyopathy

An important aspect of the clinical management of DCM is the early identification of the at-risk patient. We have shown previously that patients with TTNtv have a ∼3-fold increased odds of developing early atrial and ventricular arrhythmias compared with patients without TTNtv, highlighting how genetic information may play a role in risk stratification of patients with DCM (13). In this study, we do not detect an increased risk of long-term arrhythmic events on follow-up in patients with TTNtv DCM compared to those without. However, this finding may reflect an overall low arrhythmic risk in our cohort that has relatively mild disease. Future studies focusing on subpopulations of DCM with a higher risk of arrhythmia are therefore required to more fully elucidate a potential interaction.

We did not identify any difference in the presence of LV midwall fibrosis, a marker of adverse outcome in DCM (5), between TTNtv^+^ and TTNtv^−^ groups. In this cohort, LGE was an independent predictor of outcome in DCM, regardless of TTNtv status. This data is important as we begin to understand multimodality risk stratification in DCM (25).

### Fewer adverse outcomes in DCM as compared with registry studies

Finally, we found that the rate of adverse events in this cohort, particularly sudden cardiac death, is much lower than previous registry data (12.9% in this study compared with 21% in a registry substudy of 220 asymptomatic DCM patients) (18). Historic experience from our own institution 5, 19 is also in line with previous registry data, suggesting that our current findings are not simply a reflection of CMR bias. Optimal medical therapy in this study was in line with previously published cohorts, with over 70% of patients on beta-blocker therapy and almost 80% on angiotensin-converting enzyme inhibitor treatment 5, 18.

### Study strengths

To date, studies evaluating genotype–phenotype correlations in TTNtv DCM have differed in the presence and extent of phenotypic differences, as well as the clinical course of DCM, in patients with and without TTNtv 6, 7, 8, 9. In this study, the largest to date, we mitigate the limitations of these previous studies by using in-depth phenotyping by CMR combined with longer follow-up, and independently adjudicated clinical outcomes. This is a representative DCM cohort with respect to age, sex, and frequency of TTNtv, and the outcome predictors identified are in line with established predictors of outcome in DCM.

### Study limitations

This is a single-center study, which introduces the possibility of referral bias. However, as a major regional center for CMR, patients were initially referred from over 30 regional district general hospitals, alongside consecutive referrals from a dedicated Inherited Cardiomyopathy Service, in which CMR is performed routinely in all patients without contraindication. This broad referral base should mitigate bias.

The low arrhythmic event rate in this cohort may reflect a CMR bias, with exclusion of patients from other institutions who may not have had a CMR prior to implantable cardiac-defibrillator implantation. It is, however, possible that previous estimates of arrhythmic risk in DCM were conflated by inadvertent inclusion of patients with ischemic heart disease into echocardiography-based studies, which has been avoided through careful CMR-based phenotyping in this cohort.

These findings require replication in an independent cohort. At present, this is the largest published cohort of patients with DCM who have undergone CMR, genetic sequencing, and adjudicated long-term follow-up. However, the number of events within this cohort was not sufficient to show an effect of TTNtv on outcomes in DCM patients. With larger, multicenter studies and longer-term follow-up, the effects of TTNtv on clinical outcome may become more apparent.

## Conclusions

In this study, we identified distinct cardiac endophenotypes of TTNtv DCM that may offer mechanistic insights into the pathogenesis of DCM. We also found that the clinical outcome in TTNtv DCM is similar to patients without TTNtv DCM in a cohort of patients with mild to moderate disease. We note that the prevalence of TTNtv in severe disease in transplant-listed cases can be as high as 20%, almost double that in the current study, which remains unexplained. In summary, although TTNtv are common in DCM and may be used to confirm a genetic cause for DCM and for cascade screening, they do not appear to predict clinical outcomes in ambulant DCM cases with medium-term (∼4 year) follow-up.

Perspectives **COMPETENCY IN MEDICAL KNOWLEDGE:** TTNtv are the largest single genetic contributor to DCM. In ambulatory patients with DCM, TTNtv are associated with a blunted hypertrophic response but do not carry an increased risk of arrhythmias, heart failure, or death over a period of nearly 4 years.

**TRANSLATIONAL OUTLOOK:** Further studies are needed to define the mechanisms by which TTNtv affect outcomes in patients with DCM and to assess their impact on higher risk patients over longer-term follow-up.
