# Supplementary material for: Phenotype and Clinical Outcomes of Titin Cardiomyopathy
Source: J Am Coll Cardiol. 2017 Oct 31;70(18):2264–74. doi: 10.1016/j.jacc.2017.08.063 (PMC5666113; doi:10.1016/j.jacc.2017.08.063)
Supplement: Online Data [file mmc1.docx]

Integrated analysis of the phenotype and clinical outcomes of titin cardiomyopathy

*Genotype, phenotype and outcome study of titin DCM*

Supplementary material

Bioinformatics analysis

*Illumina sequencing data*

Demultiplexing of sequence data was performed with MiSeq/NexSeq Control software or Bcl2FastQ conversion and the FastQ files were subjected to quality control with the FastQC v.0.10.1. After low quality reads (<20) were trimmed using PrinSeq v0.20.4^5^, reads were aligned to the HG19 reference genome using BWA v0.7.10. Picard v1.115 and GATK v3.2-2 were used to mark duplicate reads and perform local realignment around indels and base quality score recalibration. Bases covered by at least 10 reads with a mapping quality ≥10 and base quality ≥20 were denoted as “callable”, i.e. adequately covered for variant calling with recommended GATK parameters. Variant calling was performed with GATK HaplotypeCaller and UnifiedGenotyper.

*SOLiD sequencing data*

The SOLiD reads were aligned in colour space using LifeScope™ v2.5.1 “Targeted re-sequencing” pipeline. The SOLiD Accuracy Enhancement Tool (SAET) was used to improve color call accuracy prior to mapping. Variants were called by the diBayes and SmallIndel packages in LifeScope software as well as GATK UnifiedGenotyper. Bases covered by at least 10 reads with a mapping quality ≥10 and base quality ≥20 were denoted as “callable”.

All detected variants from both platforms were functionally annotated using the Variant Effect Predictor (VEP v83).

Predictors of left ventricular (LV) mass

To build the baseline model (excluding TTNtv) predicting indexed LV mass (LVMi), biological and clinical variables that could plausibly predict LV mass were selected for univariable analysis. These were age, gender, a history of hypertension, race, systolic blood pressure (as measured on the day of recruitment), indexed left ventricular end diastolic volume (LVEDVi) and CMR features of left ventricular non-compaction (LVNC).

Variables significant at a p value threshold of inclusion of <0.10 were retained, and then reverse stepwise selection performed with a threshold p value for exclusion of >0.05 (Online Table 9). Previously discarded variables were added to the final model to assess for changes to the coefficients or significance threshold of retained variables. This resulted in the final model of gender, hypertension and LVEDVi.

End-point Definitions

### Cardiovascular death

Cardiovascular death was defined as death due to sudden cardiac death, heart failure, acute myocardial infarction, cerebrovascular accident, cardiovascular hemorrhage, cardiovascular procedures, or other cardiovascular causes, that is death not included in the previous categories but with a specific, known cause such as pulmonary embolus (Online Table 2).

### Heart Failure composite

The heart failure composite consisted of a heart transplant, left ventricular assist-device implantation or an unplanned heart failure hospitalization.

An unplanned heart failure hospitalization was defined as ‘an event in which the patient is admitted to the hospital with a primary diagnosis of heart failure, the length of stay is at least 24 h (or extends over a calendar date), the patient exhibits new or worsening symptoms of heart failure on presentation, has objective evidence of new or worsening heart failure, and receives initiation or intensification of treatment specifically for heart failure’(1). Notably, changes to oral diuretic therapy did not qualify as initiation or intensification of treatment(1).

### Arrhythmia composite

All ventricular arrhythmias were defined in line with the American College of Cardiology, American Heart Association and European Society of Cardiology guidelines(2).

The arrhythmia end-point was a composite of sustained ventricular tachycardia (both hemodynamically stable and unstable), ventricular fibrillation, aborted sudden cardiac death and appropriate implantable cardioverter-defibrillator (ICD) discharge.

Sustained ventricular tachycardia was defined as repetitive ventricular beats in a row lasting over 30 seconds in duration at a rate greater than 100 beats per minute (cycle length less than 600 ms), and/or requiring termination due to hemodynamic compromise in less than 30 seconds(2).

Ventricular fibrillation was defined as a rapid, usually more than 300 bpm (cycle length 180 ms or less), grossly irregular ventricular rhythm with marked variability in QRS cycle length, morphology, and amplitude(2).

Aborted sudden cardiac death was diagnosed if patients received an appropriate ICD shock for ventricular arrhythmia, or had a nonfatal episode of ventricular fibrillation or spontaneous sustained ventricular tachycardia (>30 seconds in duration) causing hemodynamic compromise and requiring cardioversion(2,3). Appropriate ICD discharge was limited to shock therapy for ventricular arrhythmias (anti-tachycardia pacing and inappropriate shocks for atrial or other arrhythmias were excluded) and all discharges were adjudicated by an Electrophysiology Cardiology Consultant.

Baseline Cox model to predict primary outcome

An optimized baseline model predicting the primary end-point was built using Cox proportional hazard modeling evaluating clinical, imaging and demographic variables. As the primary analysis was to evaluate the importance of TTNtv as a predictor of outcomes over and above existing clinical predictors, the baseline model was built without inclusion of genetic data, using purposeful variable selection (Hosmer and Lemeshow (4,5)). The p value threshold for inclusion was <0.10 (for selection of variables from univariable analysis) and for exclusion >0.05 (for exclusion of variables from multivariable analysis).

The results of univariable regression cox proportional hazard modelling of these variables for the primary end-point are shown in Online Table 8 . The variables that were significant at a threshold of p<0.10 were considered for inclusion in full model building. At this stage, any variables with potential for inclusion in the next stage of model building were reviewed for co-linearity. Left and right ventricular stroke volume were excluded due to correlation with left and right ventricular ejection fraction, as well as being subject to heart rate variability and a greater susceptibility to loading conditions compared to ejection fraction. Left ventricular end diastolic and end systolic volumes were also correlated therefore only left ventricular end systolic volume was retained as previous studies have demonstrated its prognostic importance(6,7). Left ventricular mass was excluded as it is correlated with ventricular dilation. For right ventricular indices, right ventricular ejection fraction was retained based on previous work from our group demonstrating its prognostic importance over and above right ventricular volume indices(8).

Therefore the final variables included in the full model, prior to reverse stepwise selection, were left ventricular ejection fraction, right ventricular ejection fraction, mid-wall fibrosis late gadolinium enhancement, indexed left ventricular end systolic volume, indexed left atrial volume, a family history of dilated cardiomyopathy, a history of sustained ventricular tachycardia, NYHA class, a history of hypertension, diuretic use, and ACE inhibitor use. From this, stepwise selection was performed, removing the least significant variable in turn until only significant variables were remaining (p<0.05).

This left the baseline model of left ventricular ejection fraction, indexed left atrial volume, mid-wall fibrosis late gadolinium enhancement and a history of sustained ventricular tachycardia. Each previously discarded variable was added to the model in turn. None of these variables became significant on addition to the baseline model, nor did their inclusion affect the hazard ratios or p value for the remaining variables. As left and right ventricular ejection fraction are correlated, the forced inclusion of both variables was tested. The model with just left ventricular ejection fraction had a marginally lower Akaike information criterion (AIC) compared to the model with just right ventricular ejection fraction. In the model with both right and left ventricular ejection fraction, neither variable remained significant. Therefore left ventricular ejection fraction only was retained. LVESVi, LVEDVi and LVMi were not included in the multivariate model to avoid colinearity, but separate models substituting these variables individually for LVEF showed that they too were independently associated with the primary outcome, thought with smaller effect sizes and higher p values (HR and 95% CI for primary end-point: LVESVi per 10mL/m^2^= 1.09, 1.03 to 1.16, p=0.001; LVEDVi per 10mL/m^2^= 1.07, 1.01 to 1.13, p=0.02). LVMi was not predictive of the primary outcome (HR and 95% CI; LVMi per 10g/m^2^ = 1.04, 0.94-1.15, p=0.42). The final baseline model consisted of LVEF, LAVi, LGE and ventricular tachycardia. The model was tested and there was no violation of the proportionality assumption, either for individual variables or the global model.

Supplementary Tables

Online Table 1: Titin meta-transcript details

|  | **Meta transcript** |
| --- | --- |
| Description | Inferred complete meta-transcript |
| Translation length (amino acids) | 35991 |
| Number of exons | 363 |
| Ensembl transcript | ENST00000589042 |
| Ensembl protein | ENSP00000467141 |
| Havana transcript | [OTTHUMT00000450680](http://vega.sanger.ac.uk/Homo_sapiens/Transcript/Summary?t=OTTHUMT00000450680) |
| LRG | LRG_391_t1 |

Online Table 2: Definition of cardiovascular death. Adapted from the 2014 American College of Cardiology and American Heart Association definition of cardiovascular end-points in clinical trials document(1). CV=cardiovascular, MI= myocardial infarction, HF= heart failure, ICD=implantable cardiac defibrillator.

| **Subtype of cardiovascular death** | **Definition** |
| --- | --- |
| Acute myocardial infarction (MI) | Death by any cardiovascular mechanism (arrhythmia, sudden death, heart failure, stroke, pulmonary embolus, peripheral arterial disease) within 30 d after an acute MI, related to the immediate consequences of the MI, such as progressive HF or recalcitrant arrhythmia. There may be assessable (attributable) mechanisms of cardiovascular death during this time period, but for simplicity, if the cardiovascular death occurs within 30 days of an acute MI, it will be considered a death due to MI.  Note: Acute MI should be verified to the extent possible by the diagnostic criteria outlined for acute MI or by autopsy findings showing recent MI or recent coronary thrombosis. Death resulting from a procedure to treat an MI (PCI or CABG), or to treat a complication resulting from MI, should also be considered death due to acute MI. Death resulting from an elective coronary procedure to treat myocardial ischemia (i.e., chronic stable angina) or death due to an MI that occurs as a direct consequence of a cardiovascular investigation/procedure/operation should be considered as a death due to a cardiovascular procedure. |
| Sudden cardiac death | Death that occurs unexpectedly and not within 30 d of an acute MI.  Note: Sudden cardiac death includes the following scenarios:  • Death witnessed and occurring without new or worsening symptoms  • Death witnessed within 60 min of the onset of new or worsening cardiac symptoms unless the symptoms suggest acute MI  • Death witnessed and attributed to an identified arrhythmia (egg, captured on an electrocardiographic recording, witnessed on a monitor, or unwitnessed but found on ICD review)  • Death after unsuccessful resuscitation from cardiac arrest (e.g., ICD unresponsive sudden cardiac death, pulseless electrical activity arrest)  • Death after successful resuscitation from cardiac arrest and without identification of a specific cardiac or noncardiac etiology  • Unwitnessed death in a subject seen alive and clinically stable ≤24 h before being found dead without any evidence supporting a specific noncardiovascular cause of death (information about the patient’s clinical status preceding death should be provided if available)  Unless additional information suggests an alternate specific cause of death (e.g., Death due to Other Cardiovascular Causes), if a patient is seen alive ≤24 h before being found dead, sudden cardiac death should be recorded. For patients who were not observed alive within 24 h of death, undetermined cause of death should be recorded (e.g., a subject found dead in bed but who had not been seen by family members for >24 h). |
| Heart failure | Death associated with clinically worsening symptoms and/or signs of HF, regardless of HF etiology  Note: Deaths due to HF can have various etiologies, including single or recurrent MIs, ischemic or nonischemic cardiomyopathy, hypertension, or valvular disease. |
| Stroke | Death after a stroke that is either a direct consequence of the stroke or a complication of the stroke.  Note: Acute stroke should be verified to the extent possible by the diagnostic criteria outlined for stroke. |
| CV procedure | Death caused by the immediate complication(s) of a cardiovascular procedure |
| CV hemorrhage | Death related to hemorrhage such as a non stroke intracranial hemorrhage, (e.g., subdural hematoma) nonprocedural or non traumatic vascular rupture (e.g., aortic aneurysm), or hemorrhage causing cardiac tamponade |
| CV other | Cardiovascular death not included in the above categories but with specific, known cause (e.g., pulmonary embolus) |

Online Table 3: Baseline demographics in DCM cohort.

| **Variable** | **Total cohort, n=716** |
| --- | --- |
| Age at baseline scan (years) (mean (sd)) | 53.5 (14.3) |
| Self reported race (%) |  |
| Afro-Caribbean | 26 (3.6) |
| African | 16 (2.2) |
| Asian | 38 (5.3) |
| Caucasian | 608 (84.9) |
| Chinese | 3 (0.4) |
| Mixed | 4 (0.6) |
| Other | 21 (2.9) |
| Male gender (%) | 469 (65.5) |
| NYHA class (%)  (Missing data, n=31, 4.3%) |  |
| 1 | 300 (43.8) |
| 2 | 277 (40.4) |
| 3 | 100 (14.6) |
| 4 | 8 (1.2) |
| Family history of DCM (%) | 113 (15.8) |
| Family history of sudden cardiac death (%) | 107 (14.9) |
| Moderate alcohol excess (%) * | 111 (15.5) |
| Chemotherapy (%) | 34 (4.7) |
| Inherited muscle disease (%) | 3 (0.4) |
| Peripartum cardiomyopathy (%) | 7 (1.0) |
| Beta blocker use (%) | 504 (70.4) |
| ACE inhibitor or ARB use (%) | 567 (79.2) |
| Aldosterone Antagonist use (%) | 257 (35.9) |
| Diuretic use (%) | 328 (45.8) |
| Hypertension (%) | 213 (29.7) |
| Diabetes mellitus (%) | 87 (12.2) |

* = Patients with a history of alcohol consumption in excess of UK government weekly ‘sensible limits’; Patients meeting criteria for alcoholic cardiomyopathy were excluded from the cohort.

Online Table 4: Baseline cardiac MRI phenotype in DCM cohort. Continuous data are summarized as mean ± standard deviation. Categorical data are summarized as count and percentages.

| **CMR variable** | **DCM, n=716** |
| --- | --- |
| Left ventricular ejection fraction (%) | 39.1 (12.4) |
| Indexed LV end diastolic volume (mL/m^2^) | 127.1 (35.7) |
| Indexed LV end systolic volume (mL/m^2^) | 79.9 (35.7) |
| Indexed LV stroke volume (mL/m^2^) | 47.3 (13.3) |
| Indexed LV mass (g/m^2^) | 90.4 (25.9) |
| Right ventricular ejection fraction (%) | 51.7 (13.8) |
| Indexed RV end diastolic volume (mL/m^2^) | 87.8 (24.2) |
| Indexed RV end systolic volume (mL/m^2^) | 44.0 (21.7) |
| Indexed RV stroke volume (mL/m^2^) | 43.9 (12.8) |
| CMR features of LV non-compaction | 36 (5.0) |
| Mid wall late gadolinium enhancement | 250 (34.9) |
| Bystander myocardial infarction |  |
| Yes (in patients with normal coronary artery status) | 17 (2.4) |
| Late gadolinium enhancement consistent with myocarditis |  |
| No | 656 (91.6) |
| Yes | 48 (6.7) |
| Mitral regurgitation |  |
| Mild | 233 (32.5) |
| Moderate | 77 (10.8) |
| None | 389 (54.3) |
| Severe | 15 (2.1) |
| Indexed left atrial volume (mL/m^2^) | 60.2 (25.5) |
| Maximum LV wall thickness (mm) | 9.9 (2.2) |
| Mean septal wall thickness (mm) | 7.8 (1.9) |
| Mean lateral wall thickness (mm) | 5.6 (1.6) |

Online Table 5: List of truncating variants in TTN gene (n=83). All variants annotated to titin meta-transcript ENST00000589042. Callers= U, Unified genotyper or H=Haplotype caller. PSI= percentage spliced in, a measure of splicing derived from RNAseq, is an estimate of the percentage of TTN transcripts that incorporate a given exon.

| **cDNA Variant** | **Variant type** | **Callers** | **Coverage** | **Allelic Balance** | **Confirmed** | **ExAC frequency** | **ExAC count** | **TTN exon** | **TTN band** | **TTN PSI** |
| --- | --- | --- | --- | --- | --- | --- | --- | --- | --- | --- |
| c.45852_45853delCA | frameshift | U,H | 984 | 0.5 | IGV | 0 | 0 | 248 | I-band | 100 |
| c.79539T>A | nonsense | U,H | 999 | 0.53 | IGV | 0 | 0 | 327 | A-band | 100 |
| c.55037delG | frameshift | U,H | 819 | 0.51 | IGV | 0 | 0 | 284 | A-band | 100 |
| c.83635delA | frameshift | U,H | 998 | 0.49 | IGV | 0 | 0 | 327 | A-band | 100 |
| c.45812T>G | nonsense | U,H | 999 | 0.43 | Sanger | 0 | 0 | 248 | I-band | 100 |
| c.82513delA | frameshift | U,H | 999 | 0.51 | Sanger | 0 | 0 | 327 | A-band | 100 |
| c.78507delT | frameshift | U,H | 1000 | 0.46 | Sanger | 0 | 0 | 327 | A-band | 100 |
| c.6790+1G>T | essential splice site | U,H | 1000 | 0.55 | Sanger | 0 | 0 | 29 | I-band | 100 |
| c.81262_81269delCAGATGCT | frameshift | U,H | 927 | 0.46 | Sanger | 0 | 0 | 327 | A-band | 100 |
| c.97062delA | frameshift | U,H | 604 | 0.43 | IGV | 0 | 0 | 349 | A-band | 99 |
| c.12757C>T | nonsense | U,H | 1000 | 0.51 | Sanger | 0 | 0 | 49 | I-band | 99 |
| c.55525_55531delGACAGGA | frameshift | U,H | 999 | 0.46 | Sanger | 0 | 0 | 288 | A-band | 100 |
| c.43792delG | frameshift | U,H | 1000 | 0.5 | Sanger | 0 | 0 | 238 | I-band | 100 |
| c.78184G>T | nonsense | U,H | 1000 | 0.48 | Sanger | 0 | 0 | 327 | A-band | 100 |
| c.10303+2T>C | essential splice site | U,H | 618 | 0.52 | IGV | 8.25E-06 | 1 | 44 | I-band | 100 |
| c.59226T>G | nonsense | U,H | 967 | 0.53 | IGV | 0 | 0 | 301 | A-band | 100 |
| c.101996G>A | nonsense | U,H | 644 | 0.52 | Sanger | 0 | 0 | 359 | M-band | 100 |
| c.60931C>T | nonsense | U,H | 999 | 0.52 | Sanger | 0 | 0 | 305 | A-band | 100 |
| c.98506C>T | nonsense | U,H | 1000 | 0.47 | Sanger | 0 | 0 | 353 | A-band | 100 |
| c.48527G>A | nonsense | U,H | 999 | 0.46 | Sanger | 0 | 0 | 260 | A-band | 100 |
| c.45307C>T | nonsense | U,H | 1000 | 0.47 | IGV | 0 | 0 | 246 | I-band | 100 |
| c.100644dupT | frameshift | U,H | 988 | 0.52 | IGV | 0 | 0 | 358 | A-band | 99 |
| c.50170C>T | nonsense | U,H | 502 | 0.54 | Sanger | 0 | 0 | 267 | A-band | 100 |
| c.69630C>A | nonsense | U,H | 999 | 0.51 | Sanger | 0 | 0 | 326 | A-band | 100 |
| c.52035_52036insTT | frameshift | U,H | 1000 | 0.57 | Sanger | 0 | 0 | 274 | A-band | 100 |
| c.84120delT | frameshift | U,H | 999 | 0.5 | IGV | 0 | 0 | 327 | A-band | 100 |
| c.64449dupA | frameshift | U,H | 1000 | 0.52 | IGV | 0 | 0 | 310 | A-band | 100 |
| c.58732+2T>C | essential splice site | U,H | 1000 | 0.47 | Sanger | 0 | 0 | 299 | A-band | 100 |
| c.55525_55531delGACAGGA | frameshift | U,H | 999 | 0.48 | Sanger | 0 | 0 | 288 | A-band | 100 |
| c.8307_8308delTG | frameshift | U,H | 998 | 0.45 | Sanger | 0 | 0 | 35 | I-band | 100 |
| c.63025C>T | nonsense | U,H | 999 | 0.51 | Sanger | 0 | 0 | 305 | A-band | 100 |
| c.81321C>G | nonsense | U,H | 999 | 0.47 | Sanger | 0 | 0 | 327 | A-band | 100 |
| c.50170C>T | nonsense | U,H | 636 | 0.46 | Sanger | 0 | 0 | 267 | A-band | 100 |
| c.47875+1G>A | essential splice site | U,H | 999 | 0.51 | Sanger | 0 | 0 | 256 | A-band | 100 |
| c.47697C>A | nonsense | U,H | 1000 | 0.51 | Sanger | 0 | 0 | 255 | A-band | 100 |
| c.86967G>A | nonsense | U,H | 999 | 0.49 | Sanger | 0 | 0 | 328 | A-band | 100 |
| c.67567delG | frameshift | U,H | 999 | 0.47 | IGV | 0 | 0 | 320 | A-band | 100 |
| c.4724_4728delTGAAA | frameshift | U,H | 998 | 0.48 | Sanger | 0 | 0 | 27 | near Z-disk | 100 |
| c.12643_12644delCA | frameshift | U,H | 839 | 0.52 | Sanger | 0 | 0 | 49 | I-band | 99 |
| c.92683C>T | nonsense | U,H | 528 | 0.45 | Sanger | 0 | 0 | 340 | A-band | 100 |
| c.86641delC | frameshift | U,H | 1000 | 0.46 | Sanger | 0 | 0 | 327 | A-band | 100 |
| c.93291_93301delTGTTGGTGAGC | frameshift | U,H | 1000 | 0.52 | Sanger | 0 | 0 | 340 | A-band | 100 |
| c.52223_52227dupAGAAA | frameshift | U,H | 1000 | 0.53 | Sanger | 0 | 0 | 275 | A-band | 100 |
| c.76666_76684dupATAATTGATGTCACTAGCA | frameshift | U,H | 999 | 0.45 | Sanger | 0 | 0 | 327 | A-band | 100 |
| c.100943_100944delGA | frameshift | U,H | 996 | 0.47 | IGV | 0 | 0 | 359 | M-band | 100 |
| c.51781C>T | nonsense | U,H | 1000 | 0.52 | IGV | 0 | 0 | 274 | A-band | 100 |
| c.61855delG | frameshift | U,H | 1000 | 0.52 | Sanger | 0 | 0 | 305 | A-band | 100 |
| c.6322G>T | nonsense | U,H | 1000 | 0.46 | IGV | 0 | 0 | 28 | near Z-disk / I-band | 100 |
| c.51965dupT | frameshift | U,H | 828 | 0.5 | IGV | 0 | 0 | 274 | A-band | 100 |
| c.100267_100268delAA | frameshift | U,H | 994 | 0.49 | IGV | 0 | 0 | 358 | A-band | 99 |
| c.83928dupT | frameshift | U,H | 1000 | 0.52 | Sanger | 0 | 0 | 327 | A-band | 100 |
| c.3380+1G>C | essential splice site | U,H | 341 | 0.41 | IGV | 0 | 0 | 20 | near Z-disk | 100 |
| c.47697C>A | nonsense | U,H | 721 | 0.53 | IGV | 0 | 0 | 255 | A-band | 100 |
| c.86821+2T>A | essential splice site | U,H | 717 | 0.49 | IGV | 8.91E-06 | 1 | 327 | A-band | 100 |
| c.41473C>T | nonsense | U,H | 955 | 0.49 | Sanger | 0 | 0 | 227 | I-band | 100 |
| c.53881+1G>T | essential splice site | U,H | 985 | 0.47 | Sanger | 0 | 0 | 280 | A-band | 100 |
| c.106629delA | frameshift | U,H | 999 | 0.5 | Sanger | 0 | 0 | 361 | M-band | 100 |
| c.87716delG | frameshift | U,H | 366 | 0.56 | Sanger | 0 | 0 | 330 | A-band | 100 |
| c.82240C>T | nonsense | U,H | 999 | 0.51 | IGV | 1.66E-05 | 2 | 327 | A-band | 100 |
| c.53206C>T | nonsense | U,H | 1000 | 0.45 | IGV | 0 | 0 | 278 | A-band | 100 |
| c.89216delC | frameshift | U,H | 995 | 0.54 | IGV | 0 | 0 | 335 | A-band | 100 |
| c.41447delG | frameshift | U,H | 674 | 0.5 | Sanger | 0 | 0 | 227 | I-band | 100 |
| c.89750dupG | frameshift | U,H | 999 | 0.49 | Sanger | 0 | 0 | 336 | A-band | 100 |
| c.85090C>T | nonsense | U,H | 999 | 0.52 | Sanger | 0 | 0 | 327 | A-band | 100 |
| c.102958delA | frameshift | U,H | 1000 | 0.54 | IGV | 0 | 0 | 359 | M-band | 100 |
| c.43602_43615delGCGCCTACACACCA | frameshift | U,H | 803 | 0.55 | Sanger | 0 | 0 | 237 | I-band | 100 |
| c.100943_100944delGA | frameshift | U,H | 1000 | 0.41 | Sanger | 0 | 0 | 359 | M-band | 100 |
| c.75250C>T | nonsense | U,H | 1000 | 0.47 | IGV | 0 | 0 | 327 | A-band | 100 |
| c.94721_94722delTC | frameshift | U,H | 997 | 0.49 | IGV | 0 | 0 | 342 | A-band | 100 |
| c.63025C>T | nonsense | U,H | 997 | 0.49 | IGV | 0 | 0 | 305 | A-band | 100 |
| c.94978dupG | frameshift | U,H | 999 | 0.47 | IGV | 0 | 0 | 343 | A-band | 100 |
| c.41473C>T | nonsense | U,H | 473 | 0.54 | IGV | 0 | 0 | 227 | I-band | 100 |
| c.40791dupG | frameshift | U,H | 468 | 0.49 | Sanger | 0 | 0 | 224 | I-band | 100 |
| c.63025C>T | nonsense | U,H | 998 | 0.51 | IGV | 0 | 0 | 305 | A-band | 100 |
| c.12010G>T | nonsense | U,H | 584 | 0.48 | IGV | 0 | 0 | 49 | I-band | 99 |
| c.83416C>T | nonsense | U,H | 999 | 0.5 | IGV | 0 | 0 | 327 | A-band | 100 |
| c.59201_59202delCT | frameshift | U,H | 379 | 0.55 | IGV | 8.28E-06 | 1 | 301 | A-band | 100 |
| c.73846C>T | nonsense | U,H | 680 | 0.53 | IGV | 0 | 0 | 327 | A-band | 100 |
| c.83416C>T | nonsense | U,H | 658 | 0.48 | IGV | 0 | 0 | 327 | A-band | 100 |
| c.58870C>T | nonsense | U,H | 657 | 0.48 | IGV | 0 | 0 | 300 | A-band | 100 |
| c.52254G>A | nonsense | U,H | 377 | 0.47 | IGV | 0 | 0 | 275 | A-band | 100 |
| c.44364delC | frameshift | U,H | 250 | 0.52 | IGV | 0 | 0 | 241 | I-band | 100 |
| c.55525_55531delGACAGGA | frameshift | S,U | 250 | 0.23 | Sanger | 0 | 0 | 288 | A-band | 100 |

Online Table 6: Table showing the number of patients meeting each end-point (truncated dataset). *Cardiovascular death included 18 patients with heart failure death, 3 patients with sudden cardiac death, and 1 patient each with acute myocardial infarction, cerebrovascular accident and cardiovascular –other.

|  | **Primary composite** | **Cardiovascular death*** | **Arrhythmic secondary** | **Heart Failure secondary** |
| --- | --- | --- | --- | --- |
| Yes | 78 | 24 | 24 | 50 |
| No | 526 | 580 | 580 | 554 |

Online Table 7: Composition of events leading to primary composite end-point. Patients were censored at first event.

| **CV death** | **Heart Failure hospitalization** | **Cardiac transplant** | **LVAD** | **Stable sustained VT** | **Unstable sustained VT** | **Aborted sudden cardiac death** | **Appropriate ICD activation** | **Ventricular fibrillation** | **Count (n=78)** |
| --- | --- | --- | --- | --- | --- | --- | --- | --- | --- |
| No | Yes | No | No | No | No | No | No | No | 24 |
| Yes | No | No | No | No | No | No | No | No | 12 |
| Yes | Yes | No | No | No | No | No | No | No | 8 |
| No | No | No | No | Yes | No | No | No | No | 7 |
| No | No | No | No | Yes | No | Yes | Yes | No | 3 |
| No | No | No | No | Yes | Yes | Yes | Yes | No | 3 |
| No | Yes | Yes | No | No | No | No | No | No | 3 |
| No | No | Yes | No | No | No | No | No | No | 2 |
| No | Yes | No | Yes | No | No | No | No | No | 2 |
| No | No | No | No | No | No | Yes | Yes | Yes | 1 |
| No | No | No | No | Yes | Yes | No | No | No | 1 |
| No | No | No | No | Yes | Yes | Yes | Yes | Yes | 1 |
| No | No | No | Yes | No | No | No | No | No | 1 |
| No | No | Yes | Yes | No | No | No | No | No | 1 |
| No | Yes | No | No | No | Yes | Yes | Yes | Yes | 1 |
| No | Yes | No | No | Yes | No | No | No | No | 1 |
| No | Yes | No | Yes | No | Yes | Yes | Yes | No | 1 |
| No | Yes | Yes | No | Yes | Yes | Yes | Yes | No | 1 |
| No | Yes | Yes | Yes | No | No | No | No | No | 1 |
| Yes | No | No | Yes | Yes | Yes | Yes | Yes | No | 1 |
| Yes | Yes | No | No | Yes | No | No | No | Yes | 1 |
| Yes | Yes | No | No | Yes | No | Yes | Yes | No | 1 |
| Yes | Yes | No | Yes | Yes | No | Yes | No | Yes | 1 |

Online Table 8: Results of univariable Cox proportional hazard modelling of demographic, clinical, and imaging variables predicting primary end-point. HR= hazard ratio, CI= confidence interval

| **Variable** | **Unit** | **HR** | **95% CI** | **P** |
| --- | --- | --- | --- | --- |
| ACE inhibitor /A2RB Use | Yes | 1.74 | 0.9-3.38 | 0.10 |
| Age | per 10 years | 1.09 | 0.93-1.28 | 0.3 |
| Aldosterone Antagonist Use | Yes | 1.44 | 0.92-2.23 | 0.11 |
| Atrial fibrillation | Present | 1.31 | 0.82-2.11 | 0.26 |
| Beta blocker Use | Yes | 1.39 | 0.82-2.34 | 0.22 |
| Diabetes Mellitus | Present | 1.26 | 0.69-2.31 | 0.45 |
| Diuretic | Yes | 2.1 | 1.34-3.29 | 0.001 |
| Ethnicity | Caucasian | 0.79 | 0.43-1.45 | 0.44 |
| Family history of DCM | Present | 0.47 | 0.23-0.99 | 0.05 |
| Family history of sudden cardiac death | Present | 0.64 | 0.31-1.32 | 0.23 |
| CMR features of left ventricular non compaction | Present | 0.89 | 0.28-2.81 | 0.84 |
| Gender | Male | 0.98 | 0.62-1.56 | 0.93 |
| Heart rate | Per 1 bpm | 0.99 | 0.98-1.01 | 0.40 |
| History of hypertension | Present | 1.66 | 1.06-2.61 | 0.03 |
| History of non sustained ventricular tachycardia | Present | 2.06 | 1.23-3.45 | 0.01 |
| History of ventricular tachycardia | Present | 4.7 | 2.04-10.85 | <0.001 |
| Indexed left ventricular end systolic volume | per 10mL/m2 | 1.13 | 1.08-1.19 | <0.0001 |
| Indexed left atrial volume | per 10mL/m2 | 1.11 | 1.06-1.15 | <0.0001 |
| Indexed left ventricular end diastolic volume | per 10mL/m2 | 1.11 | 1.06-1.16 | <0.0001 |
| Indexed left ventricular mass | per 10g/m2 | 1.13 | 1.05-1.21 | 0.002 |
| Indexed left ventricular stroke volume | per 10mL/m2 | 0.86 | 0.73-1.01 | 0.07 |
| Indexed right ventricular end diastolic volume | per 10mL/m2 | 1.03 | 0.94-1.14 | 0.47 |
| Indexed right ventricular end systolic volume | per 10mL/m2 | 1.16 | 1.06-1.27 | 0.001 |
| Indexed right ventricular stroke volume | per 10mL/m2 | 0.73 | 0.61-0.86 | <0.001 |
| Left bundle branch block | Present | 1.08 | 0.66-1.76 | 0.77 |
| Left ventricular ejection fraction | per 10% | 0.69 | 0.58-0.82 | <0.0001 |
| Mid wall fibrosis LGE | Present | 2.28 | 1.47-3.54 | <0.0001 |
| NYHA | Class IV | 7.93 | 1.32-47.66 | 0.02 |
| NYHA | Class III | 3.66 | 1.09-12.3 | 0.04 |
| NYHA | Class II | 1.55 | 0.47-5.08 | 0.47 |
| NYHA | Class I | 1.26 | 0.38-4.16 | 0.71 |
| Right ventricular ejection fraction | per 10% | 0.76 | 0.65-0.89 | <0.001 |

Online Table 9: Variables evaluated on univariable linear regression as predictors of indexed left ventricular mass (LVMi) to build baseline model.

| **Variable** | **Estimate change in LVMi (g/m^2^)** | **P value** | **Lower CI** | **Upper CI** |
| --- | --- | --- | --- | --- |
| Age (per 1 year) | 0.1 | 0.25 | -0.1 | 0.2 |
| Male Gender | 17.7 | <0.0001 | 13.9 | 21.5 |
| Race (compared to Afro-Caribbean) |  |  |  |  |
| African | 6.0 | 0.46 | -10.0 | 22.1 |
| Asian | -15.3 | 0.02 | -28.2 | -2.3 |
| Caucasian | -9.7 | 0.06 | -19.8 | 0.5 |
| Chinese | -6.1 | 0.70 | -36.9 | 24.7 |
| Mixed | -0.8 | 0.96 | -27.9 | 26.4 |
| Other | -8.8 | 0.26 | -24.1 | 6.4 |
| History of hypertension | 6.8 | 0.001 | 2.7 | 11.0 |
| Systolic blood pressure (per 1mmHg) | 0.0 | 0.76 | -0.1 | 0.1 |
| LVEDVi (per 1mL/m^2^) | 0.42 | <0.0001 | 0.37 | 0.46 |
| CMR features of LVNC | -3.7 | 0.42 | -12.6 | 5.3 |

Supplementary Figures


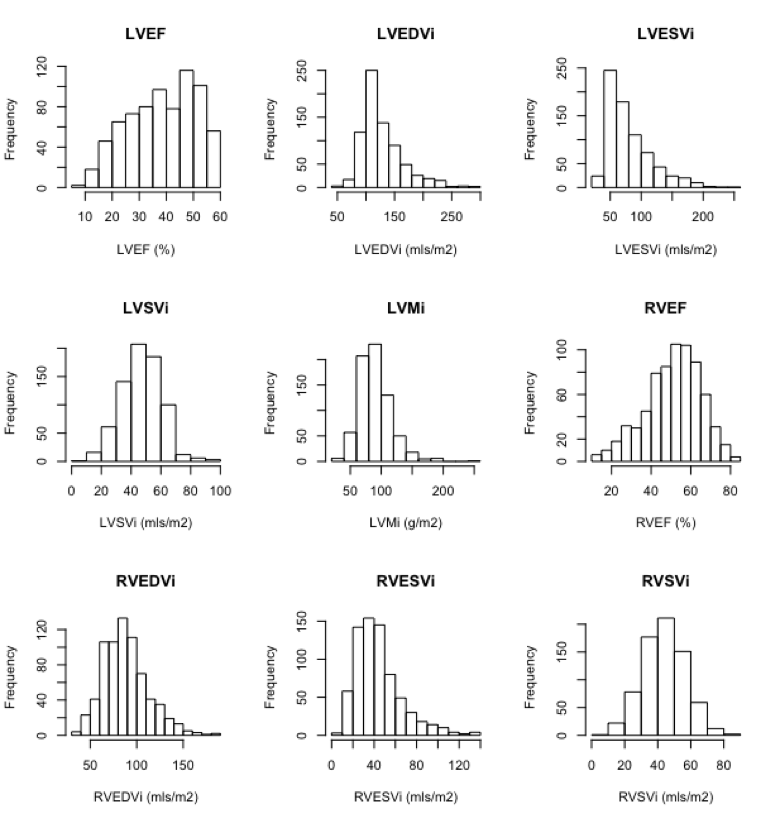


Online Figure 1: Histograms showing the distribution of left and right ventricular function and volumes, indexed to body surface area.

L/RVEF=left/right ventricular ejection fraction, L/RVEDVi=indexed left/right ventricular end diastolic volume, L/RVESVi= indexed left/right ventricular end systolic volume, L/RVSVi=indexed left/right ventricular stroke volume, LVMi=indexed left ventricular mass.

Online Figure 2: Freedom from secondary end-points stratified by TTNtv status. Curves are compared using the log rank test. **References**

1. Hicks KA, Tcheng JE, Bozkurt B et al. 2014 ACC/AHA Key Data Elements and Definitions for Cardiovascular Endpoint Events in Clinical Trials: A Report of the American College of Cardiology/American Heart Association Task Force on Clinical Data Standards (Writing Committee to Develop Cardiovascular Endpoints Data Standards). Circulation 2015;132:302-361.

2. Zipes DP, Camm AJ, Borggrefe M et al. ACC/AHA/ESC 2006 Guidelines for Management of Patients With Ventricular Arrhythmias and the Prevention of Sudden Cardiac Death: a report of the American College of Cardiology/American Heart Association Task Force and the European Society of Cardiology Committee for Practice Guidelines (writing committee to develop Guidelines for Management of Patients With Ventricular Arrhythmias and the Prevention of Sudden Cardiac Death): developed in collaboration with the European Heart Rhythm Association and the Heart Rhythm Society. Circulation 2006;114:e385-484.

3. Gulati A, Jabbour A, Ismail TF et al. Association of fibrosis with mortality and sudden cardiac death in patients with nonischemic dilated cardiomyopathy. JAMA 2013;309:896-908.

4. Hosmer Jr DW, Lemeshow S, Sturdivant RX. Applied logistic regression: John Wiley & Sons, 2013.

5. Hosmer DJ. S.. Lemeshow;(1999).‘Applied Survival Analysis. Regression Modeling of Time to Event Data.’. Wiley: New York.

6. Gold MR, Daubert C, Abraham WT et al. The effect of reverse remodeling on long-term survival in mildly symptomatic patients with heart failure receiving cardiac resynchronization therapy: results of the REVERSE study. Heart Rhythm 2015;12:524-530.

7. McManus DD, Shah SJ, Fabi MR et al. Prognostic value of left ventricular end-systolic volume index as a predictor of heart failure hospitalization in stable coronary artery disease: data from the Heart and Soul Study. J Am Soc Echocardiogr 2009;22:190-197.

8. Gulati A, Ismail TF, Jabbour A et al. The prevalence and prognostic significance of right ventricular systolic dysfunction in nonischemic dilated cardiomyopathy. Circulation 2013;128:1623-1633.
